# The timing of the *Castelnovisation* of southwestern Europe: A Bayesian modelling insight from the Romagnano Loc III rock shelter sequence (Trento, Italy)

**DOI:** 10.1371/journal.pone.0331392

**Published:** 2025-09-16

**Authors:** Salvador Pardo-Gordó, Alex Fontana, Vanessa Extrem-Membrado, Emilio Vacas-Fumero, Rossella Duches, Elisabetta Flor, Federica Fontana

**Affiliations:** 1 GISPRAYA Research Group, Departmento de Geografía e Historia, Universidad de La Laguna, San Cristóbal de La Laguna, Tenerife, Spain; 2 Research and Museum Collections Office, MUSE – Museo delle Scienze di Trento, Trento, Italy; 3 PREMEDOC Research Group, Departament of Prehistory, Archaeology and Ancient History, University of Valencia, Valencia, Comunitat Valenciana, Spain; 4 Departmento de Geografía e Historia, Universidad de La Laguna, San Cristóbal de La Laguna, Tenerife, Spain; 5 Sezione di Prehistoria, MUSE – Museo delle Scienze di Trento, Trento, Italy; 6 Sezione di Scienze Preistoriche e Antropologiche, Dip. Di Studi Umanistici, Università di Ferrara, Ferrara, Italy; Universidad de Sevilla, SPAIN

## Abstract

This paper provides a review of the Mesolithic sequence at the Romagnano loc III site through Bayesian modelling, combining the radiocarbon dates obtained in the 1970s with more recent ^14^C dates. The results suggest a chronology associated with the Castelnovian complex that predates those identified in other areas of Southwestern Europe, thus offering a new working hypothesis regarding the origin of this complex. Finally, these findings are discussed within the broader context of the origin and expansion of the Blade Trapeze Complex in Southwestern Europe indicating that the emergence of this new complex was a multifaceted process that can only be fully understood through a comprehensive global approach.

## Introduction

During the VII millennium cal. BCE, essential changes in lithic technology are recorded in the Western European Mesolithic, defined as a *revolution* [1]. Despite regional variations in the tools style and ornaments [2], this techno-typological regularisation extends throughout the western Mediterranean. These changes involve introducing pressure and indirect percussion techniques to produce regular blades and bladelets that were set up into arrowheads of geometric shapes and a wide array of trapezoidal armatures. This standardization, corresponding to the Second or Late Mesolithic [3] represents a shift to the Early Mesolithic. In addition to the debate on the possible continuity of certain elements associated with lithic production [4–6], the chronology associated with the Second Mesolithic in Southern Europe, also known as *Castelnovianisation* [7], is controversial and sometimes inconsistent due to the quality of the available radiocarbon information.

This paper focuses on the stratigraphic sequence of the Romagnano Loc III rock shelter (Trentino, North-Eastern Italy), a well-known site and one of the most important deposits of reference for the Mesolithic in Italy. In 1969, Renato Perini excavated to establish the archaeological sequence of the site, carrying out four trenches (I, II, II, IV), which allowed him to document a sequence between modern times and the Mesolithic [8]. Accordingly, Alberto Broglio in the 1970s interpreted the long Mesolithic sequence by dividing it into two main cultural complexes the Tardenoisian and Sauveterrian following the recently defined French chronology [9]. At present, the chronological sequence of Holocene hunter-gatherers in Northern Italy includes several horizons [10,11]. The Early Mesolithic (Sauveterrian) is characterised by the production of irregular lamellar blanks, flakes and microlithic armatures (*Sauveterre* points, triangles and crescents). This is followed by the Late Mesolithic (Castelnovian), defined by regular blades and bladelets productions which were transformed into different types of trapezes and a variety of tools made on lamellar blanks. In this paper, we focus our objective on the application of chronological analysis of the Mesolithic layers identified in the 1970s to evaluate the Early to Late Mesolithic Transition based on new radiometric program realised. Finally, we will place these results in the context of the Castelnovisation of Southwestern Europe.

## Materials and methods

### Romagnano Loc III rock shelter

**Fig 1:**
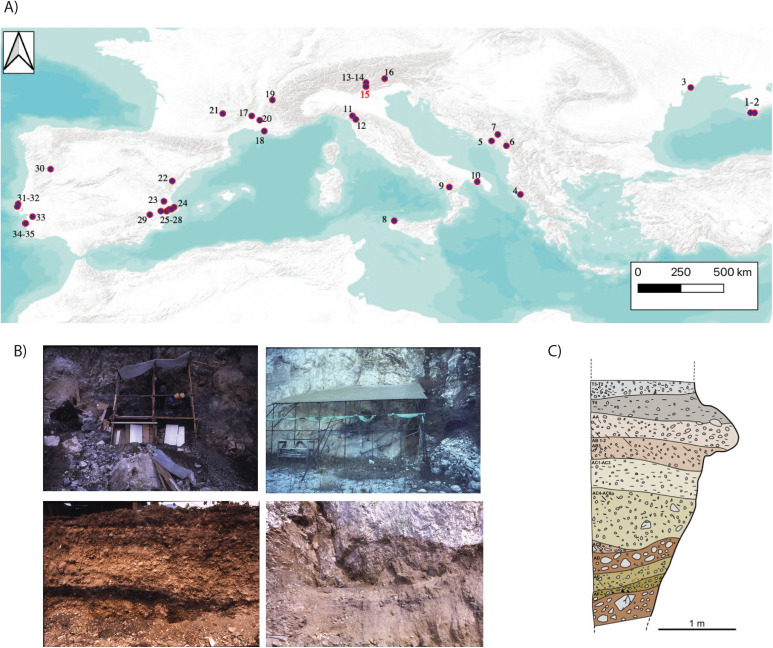
A) Sites used in this work. Crimea region: 1.Shan-Koba, 2.Lapsi 7, 3.Myrne. Western Balkans: 4.Konispol, 5.Cverna Stijena, 6.Vruća Pećina, 7.Odmut. South Italy: 8 Grotta dell’Uzzo, 9.Latronico-3, 10.Terragne di Manduria. North Italy: 11.Lama Lite, 12.Piazzana, 13.Riparo Gaban, 14.Mezzocorona-Borgonuovo, 15.Romagnano III, 16.Mondeval de Sora. France: 17.Baume Montclus, 18.Font-des-Piegons, 19.Grande-Rivoire, 20.Mourre du Sève, 21.Roquemissou. Eastern Spain: 22.Barranc de la Fontanella, 23.Cueva de la Cocina, 24.El Collao, 25.Benàmer, 26.Tossal de la Roca, 27.Abric de la Falguera, 28.Casa Corona, 29.Cueva Blanca. Portugal: 30.Prazo, 31.Costa do Pereiro, 32.Cova da Baleia, 33.Samouqueira I, 34.Vale de Romerias, 35. Vale Marim. Maps modified from ESRI World Terrain Base Map. B) General view of Romagnano excavation during 1971and profile details under CC BY license, with permission from MUSE, original copyright 1971). C) Mesolithic stratigraphy republished from [12] (redrawing by Daniel S. Hernández Hernández) under CC BY license, with permission from MUSE, original copyright 1983.

Romagnano rock shelter lies in the Adige Valley, a natural corridor between the Alps and the Po Plain (Fig 1) and contains one of the most impressive stratigraphic sequences in the Italian peninsula spanning the Mesolithic to the Iron Age. It is about 6 meters thick where the upper units correspond to Bronze Age (layer P), Chalcolithic (layer Q1), Recent Neolithic (layer R-Q2), Middle Neolithic (layer T2-S) and Early Neolithic (layer T4-T3) while the bottom ones are dated (AF-AA and U-Z) to the Mesolithic. Anthracological studies show a typical Holocene vegetation, wherein the bottom layers the *Pinus* sp. t. *sylvestris* is documented as the most abundant taxon as opposed to the dominant *Quercus* deciduous from the ceramic layers [13]. Between 1971 and 1973, A. Broglio excavated the Mesolithic layers over an approximate area of 4 meters and reaching a variable depth (from 2 to 2.5 meters). According to Broglio and Kozlowski [12] the Mesolithic layers can be broadly summarised as: 1.) **Ancient Sauveterrian**, layers AF and AE: composed by clasts and dated between 9830 ± 90 BP (R-1147) and 9420 ± 60 BP (R-1146a) which are covered by sterile alluvial gravel deposition (layer AD). From a typological view this phase is characterised by the predominance of triangles over the double back points. 2.) **Middle Sauveterrian**, layers AC9 to AC3: except from AC9 which corresponds to a transitional phase between AD and AC8, these are characterised by the predominance of fine limestone and a few detrital materials [14]. This phase is dated between 9200 ± 60 BP (R-1145) and 8590 ± 70 BP (R-1141), and the lithic industry shows a reduction in the number of segments and an increase of triangles. 3.) **Recent Sauveterrian**, layers AC2 and AC1: sedimentarily, it does not differ from the previous layers. Likewise, the lithic industry presents the same characteristics as in the Middle Sauveterrian, except for the reduction of the backed points; chronologically, this phase is located between 8560 ± 70 BP (R-1140) and 8220 ± 70 BP (R-1139). 4.) **Early Castelnovian**, layers AB3 to AB1: correspond to artificial layers characterised by the same sedimentation as the previous layers and dated by radiocarbon dating between 8140 ± 80 BP (R-1138) and 7500 ± 160 BP (R-1137a). It is at this time that the emergence of the trapezoids characteristic of the Recent Mesolithic can be observed, although some elements of the Sauveterrian tradition have been documented. Particularly AB3 is considered by the authors as a transition or a reworked layer [12]. Finally, the layers AA2-AA1 correspond to the **Late Castelnovian**. This phase has a chronology of 6480 ± 50 BP (R-1136), and although it presents some evidence of ceramics associated with taphonomy problems, the cultural information is defined by the notable increase in lamellar elements made by pressure-knapping and asymmetric Montclus type trapezes associated to symmetric ones with double concave truncations [5,6].

### The radiocarbon dates

The first chronological framework for Romagnano III was published in 1978 by the members of the Rome Radiocarbon laboratory [15]. A total of 25 conventional radiocarbon dates were obtained from singular non-identified charcoal fragments (Table 1). The radiocarbon sequence is consistent at an intra-site level. However, if we focus on the Mesolithic dates, at least the sample R-1138 (8140 ± 80 BP), recovered from the layer AB3, presents problems with the radiocarbon corpus of southwestern Europe [18]. In this sense, the first issue to be considered is the nature of the sample and the possible evidence of the *old wood effect* [19,20]. Likewise, this layer has been interpreted as a consequence of reworked [12], therefore the existence of post-depositional problems could be considered.

**Table 1:** Radiocarbon dates published previously, and the new dataset was obtained in the context of this work. All dates were calibrated in OxCal [16] using the IntCal20 curve [17].

Lab	Level	Unit	Sample	Specie	BP	SD	13C	15N	CN	Cal. 68% BCE	Cal. 95% BCE	Ref.
R-767	Layer 12	Hearth	Charcoal	n.d	2600	140	−25	n.d	–	906–540	1106–393	[15]
R-768	Land slide		Charcoal	n.d	3000	50	−25	n.d	–	1375–1128	1400–1056	[15]
R-769	P1	Burial 1	Charcoal	n.d	3720	50	−24.9	n.d	–	2198–2035	2258–1961	[15]
R-770a	P2-3	Burial 4 & 5	Charcoal	n.d	3630	50	−24.4	n.d	–	2122–1926	2189–1831	[15]
R-775	R4	Hearth	Charcoal	n.d	4810	50	−25.2	n.d	–	3643–3529	3702–3382	[15]
R-777a	T1	Hearth (under R-776)	Charcoal	n.d	5530	50	−25.5	n.d	–	4444–4339	4489–4261	[15]
R-776	T1	Hearth	Charcoal	n.d	5560	50	−25.1	n.d	–	4446–4339	4499–4331	[15]
R-779a	T2	Hearth (layer 1)	Charcoal	n.d	5470	50	−26	n.d	–	4358–4255	4445–4175	[15]
R-781	T4	Under Hearth	Charcoal	n.d	5810	50	−25.7	n.d	–	4726–4555	4790–4542	[15]
R-781a	T4	Under Hearth	Charcoal	n.d	6060	50	−26	n.d	–	5033–4853	5206–4799	[15]
CIRAM-7962	AA1	q. 3β/MUSE-PRE-c120 6131	Bone (Phalanx 2)	*Cervus elaphus*	6138	34	−21.83	3.36	3.2	5207–5002	5211–4960	This work
R-1136	AA1–2		Charcoal	n.d	6480	50	−26.9	n.d	–	5479–5377	5531–5324	[15]
CIRAM-8164	AA2	q. 5α/MUSE-PRE-c120 6142	Bone (Calcaneus)	*Cervus elaphus*	6729	36	−22.47	3.52	3.3	5669–5571	5717–5566	This work
CIRAM-10298	AB1	q. 4β/MUSE-PRE-c120 6144	Bone (Metatarsus)	*Cervus elaphus*	7106	36	−22.3	3.98	3.2	6021–5921	6063–5900	This work
CIRAM-7964	AB1	q. 3β/MUSE-PRE-c120 6129	Bone (Phalanx 1)	*Cervus elaphus*	7801	36	−21.26	2.84	3.2	6683–6593	6695–6505	This work
R-1137	AB1–2		Charcoal	n.d	7850	60	−27.3	n.d	–	6815–6597	7032–6511	[15]
CIRAM-7965	AB2	q. 4β/MUSE-PRE-c120 6128	Bone (Phalanx 1)	*Rupricapra rupricapra*	7208	36	−20.49	3.9	3.2	6080–6020	6217–5991	This work
CIRAM-10300	AB2	q. 4β/MUSE-PRE-c120 6157	Tooth (Molar 1 inf.)	*Capra Ibex*	7709	38	−21.29	4.08	3.2	6588–6481	6639–6463	This work
CIRAM-10299	AB2	q. 6β/MUSE-PRE-c120 6145	Bone (Calcaneus)	*Rupricapra rupricapra*	7977	40	−21.3	2.08	3.4	7037–6825	7046–6699	This work
R-1138	AB3	Contact with AC1	Charcoal	n.d	8140	80	−25.4	n.d	–	7314–7047	7456–6827	[15]
CIRAM-10301	AB3	q. 6β/MUSE-PRE-c120 6147	Bone (Phalanx 2)	*Cervus elaphus*	8643	40	−22.23	3.08	3.3	7711–7591	7742–7587	This work
R-1139	AC-1		Charcoal	n.d	8220	80	−26.7	n.d	–	7341–7078	7471–7061	[15]
CIRAM-7967	AC1	q. 6β/MUSE-PRE-c120 6125	Bone (Phalanx 1)	*Cervus elaphus*	8612	37	−21.69	3.61	3.3	7708–7583	7732–7581	This work
CIRAM-10302	AC2	q. 5/MUSE-PRE-c120 6148	Tooth (Molar sup.)	*Cervus elaphus*	8175	36	−22.87	4.72	3.3	7249–7073	7321–7064	This work
R-1140	AC-2		Charcoal	n.d	8560	70	−26.3	n.d	–	7649–7525	7744–7484	[15]
R-1141	AC-3		Charcoal	n.d	8590	90	−25.6	n.d	–	7722–7538	7942–7484	[15]
CIRAM-10663	AC3	q. 6α/MUSE-PRE-c120 6158	Tooth (Molar 1–2 sup.)	*Capra Ibex*	8795	35	−21.32	3.3	3.2	7946–7780	8170–7680	This work
R-1142	AC-4		Charcoal	n.d	8740	90	−26	n.d	–	7941–7606	8195–7591	[15]
CIRAM-10304	AC4	q. 5β/MUSE-PRE-c120 6150	Bone (Phalanx 3)	*Cervus elaphus*	8805	37	−23.02	3.49	3.2	7954–7769	8178–7728	This work
CIRAM-10305	AC5	q. 5β/MUSE-PRE-c120 6151	Bone (Calcaneus)	*Cervus elaphus*	8520	37	−22.12	3.61	3.3	7588–7540	7594–7526	This work
R-1143a	AC-5–6		Charcoal	n.d	9090	90	−25.7	n.d	–	8455–8232	8560–7966	[15]
CIRAM-10306	AC6	q. 6δ/MUSE-PRE-c120 6152	Bone (Calcaneus)	*Cervus elaphus*	8740	37	−22.25	3.06	3.3	7965–7611	7942–7605	This work
R-1144a	AC-7		Charcoal	n.d	9100	90	−25.5	n.d	–	8455–8239	8607–7975	[15]
CIRAM-10307	AC7	q. 6α/MUSE-PRE-c120 6153	Bone (Metatarsus)	*Cervus elaphus*	9020	37	−22.14	3.49	3.3	8281–8240	8297–8019	This work
CIRAM-10308	AC8	q. 5/MUSE-PRE-c120 6154	Bone (Metapodium)	*Sus scrofa*	9035	38	−21.53	4.57	3.3	8285–8246	8300–8211	This work
R-1145a	AC-8–9		Charcoal	n.d	9200	90	−25.9	n.d	–	8540–8301	8632–8259	[15]
R-1145	AC-8–9		Charcoal	n.d	9200	90	−26.1	n.d	–	8540–8301	8632–8259	[15]
CIRAM-10309	AC9	q. 4δ/MUSE-PRE-c120 6155	Bone (Phalanx 3)	*Cervus elaphus*	8882	37	−21.8	4.73	3.3	8197–7960	8232–7847	This work
R-1146B	AE		Charcoal	n.d	9490	80	−26.6	n.d	–	9119–8639	9186–8569	[15]
R-1146Aa	AE-1–4		Charcoal	n.d	9580	250	−25.5	n.d	–	9290–8617	9751–8292	[15]
R-1146a	AE-1–5		Charcoal	n.d	9420	60	−25.6	n.d	–	8781–8622	9117 −8490	[15]

Recently, we carried out a new radiocarbon program focused on Mesolithic layers. However, the layer that could not be dated due to the absence of collagen comes from the base of the sequence (AF/AE layer). A total of 17 new AMS dates have been obtained allowing to date the bottom of the sequence to *ca.* 9500 cal. BCE and the top of Late Castelnovian layers to *ca*. 5000 cal. BCE (Table 1). Samples were taken from animal bones (*Cervus elaphus, Rupricapra rupricapra, Sus scrofa* and *Capra ibex*) and sent to the French laboratory CIRAM; samples were treated following their standard protocols available on the laboratory website. Lastly, all dates have the information associated with stable isotopes ^13^C and ^15^N (IRMS with an error below 0,1‰) and the carbon-nitrogen ratio (CN), which is within the reference values [21,22].

### Bayesian chronological modelling

To build a high-resolution chronological framework of the Mesolithic occupations at Romagnano III and obtain the most accurate modelling possible, only short-lived dating was used, except for those layers that do not have it (AE and AF). Additionally, the available date for the Early Neolithic level of Romagnano (T4) was used as a *terminus ante quem*. We have designed several models using OxCal v. 4.4 [16], and all dates have been calibrated using the IntCal20 [17] applying a continuous sequential model and using a T general model to detect outlier dates; the dates in the model are assumed to present a Student’s t distribution with 5 degrees of freedom [23]. This proposal undertakes a continuous transition between each of the phases considered without the existence of chronological gaps. These models were conceptualised to analyse the chronology of the Sauveterrian and Castelnovian without considering their internal periodisation. This first model assumes AB3 layer as a unit associated with the Castelnovian and layer AC9 is related to the Sauveterrian. In the second model, we do not consider layers AB3 and AC9 due to their palimpsestic character, as indicated in the stratigraphic description. After exploring the Sauveterrian-Castelnovian chronology of Romagnano, we tested a sequential model that includes the internal cultural division of each internal phase of the Mesolithic sequence. This approach allows us to evaluate possible outliers statistically and to decide whether to keep or eliminate them in the construction of the chronological sequence [24]. OxCal has several diagnostic tools to assess the MCMC chain’s efficiency (convergence value) and the concordance of data (A_model_ and A_overal_ indices). For modelling to be statistically acceptable, the convergence value (C) must be higher than 95%, while the concordance values have a minimum threshold of 60%.

## Results

The first model generated (model 1 in Table 2) includes all the layers and dates considered in the methodological section. The result presents a concordance index below 60% (A_model _= 6.7%), showing the presence of inconsistencies between the radiometric data and the internal organisation of the phases (36.3% of the sample available for Romagnano are considered outliers). Therefore, a second Bayesian model (model 2 in Table 2) has been elaborated in which the two dates from the layers considered as reworked or altered have been discarded.

**Table 2:** Values obtained from the different Bayesian models generated. The code for the different OxCal models is available in Supporting Information (S1–S7 Files).

Bayesian model	Amodel	Aoverall
Model 1 (3 phases)	6.7	3.4
Model 2 (3 phases)	21.8	12.5
Model 3 (3 phases)	107.7	107.2
Model 4 (6 phases)	18.5	11.7
Model 5 (6 phases)	41.5	27.5
Model 6 (6 phases)	56.2	80.6
Model 7 (6 phases)	107.4	105.6

Although the concordance results of this model increase, they are still below the acceptance threshold.

**Fig 2:**
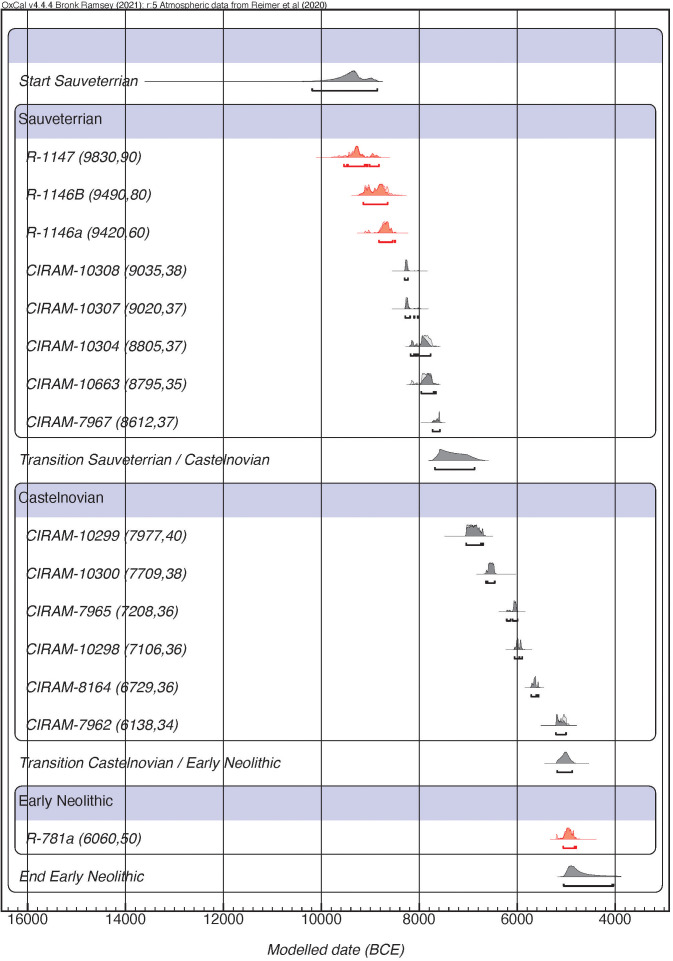
Bayesian model without considering the internal periodisation of each phase of the Mesolithic. The red colour indicates the radiocarbon dates made on charcoal.

According to the previous model, a third proposal has been made (model 3 in Table 2) removing all dates indicated as outliers (Layer AB1: CIRAM-7964; Layer AC2: CIRAM-10302; Layer AC5: CIRAM-10305 and Layer AC6: CIRAM-10306) revealing a good agreement (A_model _= 107.7%). This model allows us to have the first intra-site chronological proposal associated with the two technocomplexes explored in this work (Fig 2). Based on the results of this model, the beginning of the Sauveterrian falls between 10191 and 8859 cal. BCE (95% probability), while the transition between this latter and the Castelnovian between 7684 and 6872 cal. BCE, at 95% probability. Finally, the first evidence of agropastoral societies in Romagnano is placed between 5188−4878 cal. BCE.

**Fig 3:**
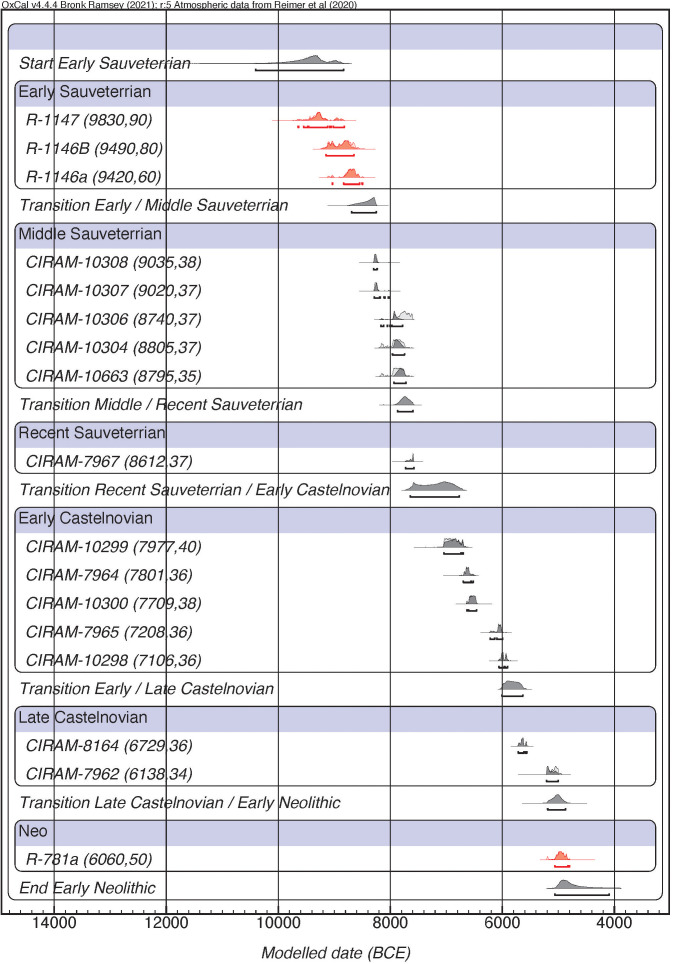
Bayesian model considering the internal periodisation of each phase of the Mesolithic. The red colour indicates the radiocarbon dates made on charcoal.

Once the first chronometric proposal was obtained, a second group of Bayesian models was made to explore the chronology of the different sub-phases according to the evolution of lithic production. This model (model 4 in Table 2) has been constructed using the same data and layers as model 1. Again, the results present a poor agreement index (A_model _= 18.5%) as model 1. So, the same procedure has been carried out as in the previous case: we have built another model removing layers AC9 and AB3 (model 5 in Table 2). Although the concordance value of this model increases (A_model _= 41.5%), it is still below the acceptance threshold. Results indicate the existence of 4 outliers, 2 of which are associated with the Middle Sauveterrian (AC6 & AC5) and the other two correspond to the two samples from the Recent Sauveterrian (AC2 & AC1). However, to avoid discarding the whole sample available for the Recent Sauveterrian, we decided to perform another Bayesian model (model 6 in Table 2), removing only those dates with outlier values greater than 60/100 (Layer AC5: CIRAM-10305). Although this model only identifies one date as a possible outlier (layer AC2: CIRAM-10302) results still show an agreement index below the threshold considered by OxCal. According to the previous model, a new model has been defined (model 7 in Table 2) removing the radiocarbon date from Layer AC2 (CIRAM-10302, 8175 ± 36 BP) and revealing a good agreement (A_model _= 107.4%). This model allows us to define better the periodisation of the Sauveterrian and the Castelnovian complexes (Fig 3).

Following the results obtained in this model (Table 3), the first evidence of the Early Sauveterrian complex starts between the second half of the X millennium cal. BCE and the first quarter of the IX millennium cal. BCE. The boundary between Early-Middle Sauveterrian encompasses the second half of the IX millennium cal. BCE (8693−8249 at 95% of probability). As far as the Middle/Late transition is concerned, it is located in the first half of the VIII millennium cal. BCE. The first evidence of trapezes marks the starting point of the Early Castelnovian (last quarter of the VIII millennium cal. BCE. to the beginning of the VI millennium cal. BCE). Finally, the reduction of triangles and the notable increase of trapezes is the turning point to differentiate the Early and Late Castelnovian. According to the Bayesian modelling obtained the transitional phase spans the first half of the VI millennium cal. BCE.

**Table 3:** Results of Romagnano rock shelter based on model 7 showing posterior (modelled) radiocarbon distributions.

*Phase*	*cal. BCE 68% (unmodelled)*	*cal. BCE 95% (unmodelled)*	*cal. BCE 68% (modelled)*	*cal. BCE 95% (modelled)*
Early Sauveterrian	9447−8622	9740−8490	9387−8624	9645−8494
Transiton Early-Middle Sauveterrian			8493−8264	8693−8249
Middle Sauveterrian	8285−7780	8300−7680	8287−7753	8299−7721
Transiton Middle-Recent Sauveterrian			7811−7656	7872−7596
Recent Sauveterrian	7708−7583	7732−7581	7653−7582	7730−7578
Transiton Sauveterrian – Castelnovian			7599−6850	7643−6771
Early Castelnovian	7037−5921	7046−5900	6971−5925	7042−5906
Transtion Early-Late Castelnovian			5941−5706	6006−5636
Late Castelnovian	5559−5002	5571−4960	5667−5038	5718−5004
Transition Castelnovian – Neolithic			5108−4942	5191−4874
Neolithic sensu lato	5033−4853	5206−4799	5011−4851	5064−4800

## Discussion

The new radiometric corpus carried out in Romagnano Loc III provides interesting insights into the *Castelnovisation* of Southwestern Europe. Firstly, our data support an early radiocarbon date for the beginning of the Castelnovian occupations in Romagnano (layer AB2 with 66% of trapezes) at the beginning of the VII millennium (7046−6747 cal. BCE). This data is consistent with Clark’s original proposal [25] and subsequent revisions for an East-West gradient of this techno-complex [1,18,26]. However, the results obtained at Romagnano suggest the need to revisit the debate concerning the pathways of expansion of this phenomenon in Southwestern Europe. In this regard, although the influence of climatic events as the primary driver of dispersal remains an open question [27], no clear correlation between the spread of the Late Mesolithic and climatic fluctuations has been identified in the geographical area examined in this work [28]. Consequently, the discussion will focusses on issues of chronology and stratigraphy (see S8 Text and S9 Table).

Some authors have suggested that pressure-knapping blade production is an original marker, and they identified a relationship between trapezes and the pressure-knapping technique in the Upper Capsian industries of the Maghreb [29]. The French school advocated this relationship could indicate a link between ‘Castelnovian-like’ industries and the Upper Capsian. Therefore, they have proposed an origin of the Blade-Trapeze industries in Sicily according to the stratigraphy of Grotta dell’Uzzo (Sicily) and the distribution of pressure-knapping blade production linked to the Late Mesolithic trapeze production in the Western Mediterranean, along with a critical review of the radiocarbon information and lithic collection from Northwestern Africa [18,30,31]. So, could Grotta dell’Uzzo be the origin of this technological complex? Despite the important research in the cave, the most reliable published Castelnovian information comes from Trench F (layers 11–14) [32,33], and the chronology is placed at the beginning of the VII millennium cal. BCE. However, the oldest date associated with the Castelnovian levels comes from an unidentified charcoal sample [34] (P-2734, 7044−6640 cal. BCE). The available short-lived dates come from human remains (OxA-V-2364-43, 6503−6222 cal. BCE) and a cetacean (MAMS-16238, 6444−6011 cal. BCE) [35] and they show a chronology that places the Castelnovian evidence from Grotta dell’Uzzo in the second half of the VII millennium cal. BCE. The results should be treated with caution because the samples may be affected by both the reservoir and the old wood effects, as widely discussed in the archaeological literature [19,34–38] but see also see S8 Text and S9 Table]. Similarly, the results of the CIMO project indicate that an early chronology for the Castelnovian levels at Grotta dell’Uzzo can be rejected [39].

The alternative theoretical origin of the Blade-Trapeze Complex was identified on the Crimean Peninsula [25,40]. According to archaeological records, some sites such as Shan-Koba [41], Lapsi7 [42], and Myrne [43], among others, are associated with the production of trapezes from the Late Mesolithic. However, while there is clear evidence that geometric arrowheads were made from blades, there are no documented instances of pressure-knapping being employed for their manufacture [44]. To this must be added the existence of confusion in the cultural attribution of the lithic assemblages due to methodological problems between typology and functionality related to the ‘kukrek inserts’ [45]. If we focus on radiometric information from the region, different radiocarbon dating programmes have been carried out in recent years [46–48]. According to the radiocarbon dates available for Myrne, the chronology of the trapezes is placed at the second part of the VIII cal. BCE. It corresponds to an open-air site with horizontal stratigraphy and is one of the most impressive sites in the region [49]. Furthermore, the radiometric information comes from several areas where trapezes are less than in other areas [47], with end scrapers being the most common tools [43]. Therefore, the information should be taken with caution, not only because of the possible existence of palimpsests but also because of the possibility that the radiocarbon dates come from a previous Mesolithic occupation based on the lithic assemblages recovered. The site of Lapsi7 is complex not only because of its stratigraphy but also because of the existence of possible levels characterised by palimpsests due to the “cohabitation of different cultural groups”, as recently highlighted by Telegin and coll. [42]. To this must be added the chronological uncertainty of layer D due to the inconsistency of the radiocarbon dates obtained. If we focus on the two dates obtained by the AMS method [41], the first evidence of trapezes is between 7745–7582 cal. BCE. Although the two dates are chronologically consistent, both have been made on charcoal remains (*Ulmus* and *Pomoideae*), so doubts about whether they are affected by the old wood effect are present. Finally, the Shan Koba shelter is the last key site to support the Crimean hypothesis. In this site, excavated between 1928 and 1936, six archaeological levels were identified, of which the upper ones (levels 1–3) have yielded trapezoidal arrowheads. While levels 1 and 2 show a Neolithic root [50], level 3 has traditionally been related to the Tardenoisian complex [51]. This Late Mesolithic level is characterised by notched bladelets, backed points and geometric microliths including trapezes, triangles and lunates [52]. Two of the five radiocarbon short-lived dates available for layer 3 should be considered as outliers [41]. The first of these comes from the deepest spit of the layer, but it has an extremely recent chronology compared to the rest (GrA-50242, 7075 ± 45 BP). Conversely, the second outlier comes from the same spit and presents an older chronology (KIA-9571, 8357 ± 52 BP) than the rest of the available dates, showing an internal coherence. In light of the existing radiometric contradiction of level 3 (split 3), a cautious approach is recommended regarding the ancient chronology of the Shan-Koba site concerning the trapeze industries.

Then, it would be reasonable to enquire whether any additional evidence might assist in investigating the Castelnovian origin. In this respect, analysing ancient DNA could be a useful avenue of inquiry. The archaeogenetics literature on hunter-gatherer groups has identified two supra-regional metapopulation groups: Eastern and Western Hunter-Gatherer clusters [53]. Nevertheless, other studies conducted in southwestern Europe, particularly in Sicily, indicate the presence of an Eastern Hunter-Gatherer signal during the Castelnovian period. This signal is associated with hunter-gatherer groups from diverse geographical regions such as Ukraine, the Iron Gates (Romania) or Northern Europe [54]. These results suggest the potential for Eastern European populations to have spread in relation to the blade-trapeze complex. Despite the lack of definitive conclusions, available data suggest the possibility of demographic expansion of the Castelnovian complex, with a potential origin in the Crimean area.

**Fig 4:**
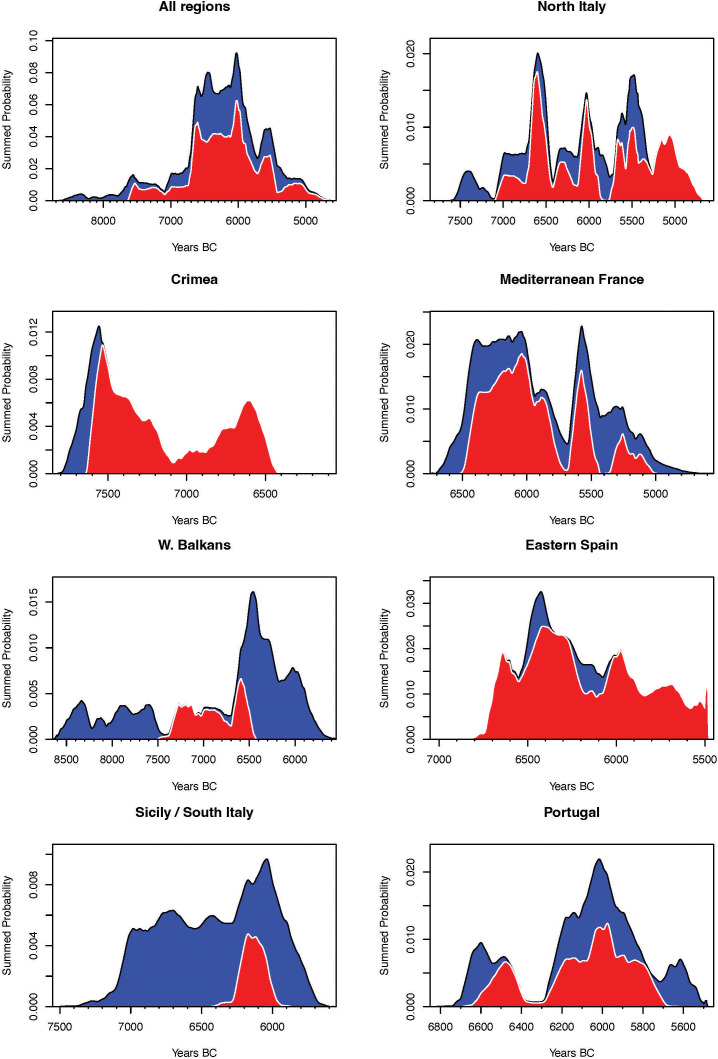
Summed probability distribution across the various geographical regions considered based on the nature of the dated sample (blue denotes all radiocarbon data; red means short-lived samples). This has been created using the R software [59] and the rcarbon package [60].

The combination of chronological and paleogenetics evidence and the available data from Romagnano and other key sites, including Cueva de La Cocina [55], Baume Montclus [56], Roquemissou [57], along with the systematic review carried out for the Western Balkans [58], allows an examination of the chronology of the earliest evidence for Castelnovian industries in southwestern Europe to be examined and the robustness of the hypotheses concerning their origin to be assessed (Fig 4).

As outlined above, the information available for the Crimean area is inconclusive. However, although there is no evidence of pressure knapping, there is no doubt about the blade nature of the trapezoidal arrowheads. Also, the different radiocarbon dates available from several archaeological sites suggest a chronology associated with the first evidence of ‘Castelnovian-like’ trapezes around the first half of the VIII millennium cal. BCE (7734−7582 cal. BCE). Technological studies concentrating on the knapping systems and new excavations with rigorous stratigraphic control and radiocarbon programs are fundamental to reinforcing this proposal.

One of the best known sites attributed to the Castelnovian in the Adriatic-Ionian zone is Konispol Cave [61]. However, the reliability of the context and cultural attribution has been questioned [62,63], and the precise chronology of this site remains uncertain, as the dating of several unidentified charcoal samples has yielded inconsistent results [61]. In the Eastern Adriatic, the most reliable information on the Late Mesolithic comes from present-day Montenegro. Late Mesolithic occupations characterised by blade and trapeze industries attributable to the Castelnovian are documented at Crvena Stijena, Vruća Pećina and Odmut [7,64,65]. Castelnovian elements include the laminar character of these industries, the use of pressure flaking and indirect percussion, and the presence of characteristic tool types, such as trapeze microliths (potentially produced in some cases with the microburin technique), notched blades, and circular endscrapers. The probable use of both knapping techniques, alongside certain technological procedures evidenced on the proximal parts of blades makes the Montenegrin *chaîne opératoire* resemble those observed in southern Italy [58].

Despite recent radiometric programmes, the chronology associated with the Castelnovian complex is still unclear. Firstly, the recently proposed dates for the Late Mesolithic levels at the Crvena Stijena indicate that the evidence for this cultural complex can be dated in the second half of the IX millennium cal. BCE (8226−7829 cal. BCE). Nevertheless, the sample from a deer metacarpus is of a date that could be considered somewhat out of context, having been derived from an association of layers determined from depths and descriptions made in the 1950s [66]. In addition, radiometric data is available from other sites, such as Odmut, Vruća and Vrbička [67]. In the case of Vrbička cave, which has a calibrated chronology between 7073−6653 cal. BCE, the lithic assemblage fails to display the characteristic attributes traditionally associated with the Castelnovian. However, blades, bladelets, and prismatic cores are reported [67]. In the case of Odmut and Vruća, the radiocarbon dates have been obtained from the bone industry (harpoons) and span a chronological range from the end of the VIII to the beginning of the VII millennium cal. BCE. Nevertheless, despite the potential association of the lithic industry with the Castelnovian complex, further evidence is required to ascertain whether the earliest dates related to the harpoons are indeed attributable to an earlier cultural horizon or not [58,68].

Moving to Italy, most evidence concerning the Castelnovian period in the southern area comes from Grotta dell’Uzzo (Sicily), Latronico 3 (Basilicate) and Terragne di Manduria (Apulia). The lithic assemblages are characterised by symmetrical/asymmetrical trapezes based on the microburin technique and the preferential use of specific raw materials to produce bladelets [33,69]. In light of the chronological reviews in Sicily [63], it is indisputable that the chronology of the Castelnovian complex started in the second half of the VII millennium cal. BCE. Regarding the southern Italian peninsula, archaeological data are more complex due to the coexistence of diverse techno-cultural traditions and a radiometric framework derived from unidentified charcoal samples [70]. In this sense, the first Castelnovian evidence in the area, according to the chronology available for the site of Latronico 3, is dated to the first half of the VII millennium cal. BCE while Terragne’s data suggest a chronology between the end of the VII millennium cal. BCE and the beginning of the VI millennium cal. BCE [71]. In the northern Apennine region, the most substantial evidence of Castelnovian activity is observed at Piazzana and Lama Lite sites [72,73]. A systematic study of the lithic industry has been conducted on both sites, which exhibit all the defining characteristics of the Castelnovian complex. However, the chronology is not solid, as the Late Mesolithic levels have been dated from unidentified charcoal samples. In this regard, while the date of Lama Lite’s context suggests a very late chronology (first half of the VI millennium cal. BCE [74]), the date of Piazzana indicates the presence of the Castelnovian in the region during the second half of the VII millennium cal. BCE [75]. Further research is required to enhance the understanding of the Castelnovian complex and establish a chronology that meets the established radiometric standards. In the case of north-eastern Italy, the information associated with the Castelnovian comes from various sites such as Riparo Gaban, Mondeval de Sora, Mezzocorona-Borgonuovo, and Romagnano among others [9]. In the case of Gaban [76], the archaeological record related to the Castelnovian layers is characterised by a high geometric component, achieved through the microburin technique. Radiometric data place the first evidence of trapezes between 7045−6467 cal. BCE, although all of these have been obtained from unidentified charcoal samples. Regarding the burial of Mondeval de Sora [77], the lithic assemblage, characterised by the presence of indirect percussion and pressure flaking, suggests a Castelnovian chronology. This has been corroborated by the radiometric analysis of the human remains, which indicates a Late Mesolithic occupation it the second half of the VII millennium cal. BCE. Another burial that provides evidence of Castelnovian presence in the region is Mezzocorona-Borgonuovo [78]. However, the radiometric data are contradictory. Recently, a new radiometric analysis was conducted following all quality standards [79], and its results suggest a chronology between 5612–5409 cal. BCE. Nevertheless, there is no lithic evidence available to conform and/or refine this chronology, especially considering that the dating obtained from the possible grave goods associated with the burial shows a chronology related to the beginning of the VII millennium cal. BCE. Finally, the chronological revision of Romagnano III places the first Castelnovian evidence at the end of the VIII millennium cal. BCE and the beginning of the VII millennium cal. BCE based on the oldest date related to layer AB2. Although younger dates have been obtained from the same stratigraphic layer, they come from a different spatial unit within that layer and can therefore be considered as reflecting a later occupation associated with the same phase of this technocomplex. This chronology is contemporary with the eastern Adriatic area and a few centuries older than that of the rest of the western Mediterranean. Consequently, an alternative origin of the Castelnovian in the Alpine area could be considered. Nevertheless, despite surpassing all radiometric quality standards such as carbon-nitrogen ratio, this date is older than others in the region. Consequently, this proposal should be regarded as a working hypothesis to be checked by further analysis and by increasing the dates for this archaeological context following Bayliss and Marshall’s proposal [80]. This will allow us to either confirm or refute this hypothesis, especially given the absence of radiocarbon dates from other regional sites, such as Vatte di Zambana and Riparo Padestel, that meet all established radiometric hygiene protocols.

In Mediterranean France, Font-des-Pigeons [81], Mourre-du-Sève [82], Montclus [83], Roquemissou [84], and other sites provide information on the Castelnovian complex. Various works that have focused on techno-typological studies have identified all the elements that define the Castelnovian, including pressure knapping, indirect percussion, prismatic cores, blades, bladelets and geometric armatures such as symmetrical and asymmetrical trapezes [85,86]. In this sense, the first evidence of this technocomplex, according to the stratigraphic and radiometric revisions in the region [56,85,87], is located in the second half of the VII millennium cal. BCE.

As far as the Iberian Peninsula, there is a wealth of information on the Late Mesolithic [88]. On the one hand, in the eastern quadrant, the most representative site, which has recently undergone a thorough revision, is the Cueva de la Cocina [55,89]. Based on its chronostratigraphic information, the Castelnovian horizon is dated to the second half of the VII millennium cal. BCE. It should be noted that at the base of the sequence, there is a date that places the first occupation of the cave in the first half of the VII millennium cal. BCE, but the associated lithic information is scarce and not very diagnostic, so it cannot be associated with the Castelnovian complex. On the other hand, in the west of the Iberian Peninsula (namely Portugal), there is a great amount of information related to the funerary practices and the way of life of Late Mesolithic groups [90]. However, the cultural information and radiocarbon data cannot be linked because most of it comes from excavations in the late 19^th^ and early 20^th^ centuries, when there was no exhaustive stratigraphic control. In this region, the context with a reliable stratigraphy that can be linked to the material culture is the Costa do Pereiro site [91], whose level 1b is associated with an industry characterised by the presence of typically Castelnovian geometric microliths (trapezes) dated to the last quarter of the VII millennium cal. BCE.

## Conclusions

The number of Late Mesolithic archaeological contexts that have undergone a systematic study of the lithic assemblages and a reliable radiometric framework remains limited. Furthermore, there is a certain degree of contradiction in some cases. Nevertheless, the chronostratigraphic review carried out at the Romagnano Loc III site allows us to revisit an alternative hypothesis regarding the origin and expansion of the Castelnovian complex. In this regard, in addition to the traditional hypothesis proposed by Gimbutas and Clark during the first half of the twenty century regarding the initial expansion from the Pontic region, an alternative origin can be proposed in the Northern Italy. This alternative hypothesis suggests that the expansion occurred in less than 500 years throughout the western Mediterranean. Nevertheless, this proposal should be regarded as a preliminary hypothesis to be further investigated in the future, given the existence of divergent data, such as that concerning the eastern Adriatic.

To conclude, the evidence indicates that the emergence of the Castelnovian was a multifaceted process that can only be fully understood through a comprehensive, global approach. While this paper has focused primarily on chronological data, strengthening our conclusions will require the future integration of additional variables, including economic, technological, funerary, and settlement patterns. Two fundamental questions remain to be addressed: first, whether the phenomenon was characterised by a unifocal origin or by a process of equifinality involving multiple centres of innovation; and second, whether the rapid spread of the Castelnovian across Europe—within less than a millennium and at a faster rate than the dispersal of agriculture, according to radiocarbon dates—suggests that demic and/or cultural diffusion processes were already at play prior to the Neolithic and it is therefore essential to identify the specific nature of these diffusion mechanisms.

## Supporting information

S1 File Oxcal Model 1. (DOCX)

S2 File Oxcal Model 2. (DOCX)

S3 File Oxcal Model 3. (DOCX)

S4 File Oxcal Model 4. (DOCX)

S5 File Oxcal Model 5. (DOCX)

S6 File Oxcal Model 6. (DOCX)

S7 File Oxcal Model 7. (DOCX)

S8 Text Radiometric quality control applied to explore the radiocarbon dates used in this work. (PDF)

S9 Table Radiocarbon dates used in this paper. (PDF)
