# Supplementary material for: The timing of the Castelnovisation of southwestern Europe: A Bayesian modelling insight from the Romagnano Loc III rock shelter sequence (Trento, Italy)
Source: PLoS One. 2025 Sep 16;20(9):e0331392. doi: 10.1371/journal.pone.0331392 (PMC12440202; doi:10.1371/journal.pone.0331392)
Supplement: S3 File — (DOCX) [file pone.0331392.s003.docx]

Plot()

{

Outlier_Model("General",T(5),U(0,4),"t");

Sequence()

{

Boundary("Start 1");

Sequence("Sauveterrian")

{

R_Date("R-1147",9830,90)

{

Outlier("General", 0.05);

};

R_Date("R-1146B",9490,80)

{

Outlier("General", 0.05);

};

R_Date("R-1146a",9420,60)

{

Outlier("General", 0.05);

};

R_Date("CIRAM-10308",9035,38)

{

Outlier("General", 0.05);

};

R_Date("CIRAM-10307",9020,37)

{

Outlier("General", 0.05);

};

R_Date("CIRAM-10304",8805,37)

{

Outlier("General", 0.05);

};

R_Date("CIRAM-10663",8795,35)

{

Outlier("General", 0.05);

};

R_Date("CIRAM-7967",8612,37)

{

Outlier("General", 0.05);

};

};

Boundary("Transition 1/2");

Sequence("Castelnovian")

{

R_Date("CIRAM-10299",7977,40)

{

Outlier("General", 0.05);

};

R_Date("CIRAM-10300",7709,38)

{

Outlier("General", 0.05);

};

R_Date("CIRAM-7965",7208,36)

{

Outlier("General", 0.05);

};

R_Date("CIRAM-10298",7106,36)

{

Outlier("General", 0.05);

};

R_Date("CIRAM-8164",6729,36)

{

Outlier("General", 0.05);

};

R_Date("CIRAM-7962",6138,34)

{

Outlier("General", 0.05);

};

};

Boundary("Transition 2/3");

Sequence("Early Neolithic")

{

R_Date("R-781a",6060,50)

{

Outlier("General", 0.05);

};

};

Boundary("End 3");

};

};
