# Supplementary material for: The timing of the Castelnovisation of southwestern Europe: A Bayesian modelling insight from the Romagnano Loc III rock shelter sequence (Trento, Italy)
Source: PLoS One. 2025 Sep 16;20(9):e0331392. doi: 10.1371/journal.pone.0331392 (PMC12440202; doi:10.1371/journal.pone.0331392)
Supplement: S8 Text — (PDF) [file pone.0331392.s008.pdf]

Supporting Information

The timing of the Castelnovisation of southwestern Europe: a Bayesian modelling insight from the Romagnano Loc III rock shelter sequence (Trento, Italy)

Salvador Pardo-Gordó, Alex Fontana, Vanessa Extrem-Membrado, Emilio Vacas-Fumero, Rossella Duches, Elisabetta Flor, Federica Fontana

This section presents the radiometric screening employed for assessing the reliability of the datings, from the perspective of sample quality and stratigraphic criteria.

1. Radiometric hygiene

The use of radiocarbon information requires defining protocols focused on radiometric hygiene, to prevent background noise into the already uncertain radiocarbon method. In this regard, since Waterbolk's pioneering work [1], several proposals have been put forward concerning the reliability of radiocarbon dates, particularly concerning the European Neolithic,[2,3 among others]. In this paper, a system rank is established based on previous proposals [4,5], organising radiometric data into seven categories, with the first category being the most reliable.

- **Rank 1**: Dates made on short-lived samples and singular elements. In the case of samples taken from bone, this category only considers those that have quality indices (isotope values and carbon-nitrogen ratio).
- **Rank 2**: Dates made on singular bone samples that lack the quality indices outlined in the previous rank. This category includes human bones provided the percentage of mire diet is known.
- **Rank 3**: Dates made on bulk short-lived samples regardless of whether the species has been identified.
- **Rank 4**: Dates made on singular long-lived samples with taxonomic identification.
- **Rank 5**: Dates made on bulk long-lived samples with taxonomic identification.
- **Rank 6**: Dates made on singular/bulk short-lived samples that require the application of a reservoir effect (fish, shell, terrestrial gastropods).
- **Rank 7**: Dates made on long-live (singular/bulk) with taxonomic identification or lacking it. This category also includes dates made on sediment.

- **Rank 8**: Dates that should not be considered under any circumstances. Dating with quality indices outside the range established by De Niro [6] and Van Klinken [7] and dates obtained from laboratories of questionable quality [8].

2. **Stratigraphic Criterion**

Parallel to the classification of ^{14}C information according to radiometric hygiene criteria, three levels of stratigraphic reliability have been established.

- **Level 1**: Stratigraphically coherent datings, both from a stratigraphic positioning perspective and a cultural point of view.
- **Level 2** refers to the archaeological levels that exhibit a cultural characterisation of Castelnovian affiliation *sensu lato*, although they present some stratigraphic inversion. This category also includes all samples associated with human remains, except those that clearly show a distinct stratigraphy and are associated with cultural information.
- **Level 3**: All datings classified in this category lack stratigraphic reliability. In this sense, this group includes radiometric dates with an incoherent stratigraphic position, radiocarbon data with unknown stratigraphic information, and archaeological levels that lack diagnostic information associated with the Castelnovian.

References

1. Waterbolk HT. Working with Radiocarbon dates. Proceedings of the Prehistoric Society. 1971;37: 15–33. doi:10.1017/S0079497X00012548

2. Zilhão J. The spread of agro-pastoral economies across Mediterranenan Europe: a view from the far west. Journal of Mediterranean Archaeology. 1993;6: 5–63. doi:10.1.1.456.9908

3. Bernabeu J. Una visión actual sobre el origen y difusión del Neolítico en la Península Ibérica. In: García Puchol O, Aura Tortosa JE, editors. 8000 años de ocupación humana en la cabecera del río de Alcoi. Alcoi: Museu d'Alcoi; 2006. pp. 189–211.

4. Pardo-Gordó S, González Marrero M del C, Vidal-Matutano P, Rodríguez Rodríguez A del C. Dataciones de contextos aborígenes y coloniales de la isla de Gran Canaria: una propuesta de protocolo de higiene radiocarbónica. Tabona: Revista de Prehistoria y Arqueología. 2022;22: 217–242. doi:10.25145/j.tabona.2022.22.11

5. Zilhão J. Time Is On My Side. In: Hadjikoumis A, Robinson E, Viner S, editors. The dynamics of neolithisation in Europe Studies in honour of Andrew Sherratt. Oxford: Oxbow Books; 2011. pp. 46–65.

6. De Niro MJ. Postmortem preservation and alteration of in vivo bone collagen isotope ratios in relation to palaeodietary reconstruction. Nature. 1985;317: 806–809. doi:doi.org/10.1038/317806a0

7. Van Klinken GJ. Bone collagen quality indicators for palaeodietary and radiocarbon measurements. Journal of Archaeological Science. 1999;26: 687–695. doi:10.1006/jasc.1998.0385

8. Blackeslee DJ. Reassessment of some radiocarbon dates from the central plains. Plains anthropologist. 1994;39: 203–210.
