# Supplementary material for: The timing of the Castelnovisation of southwestern Europe: A Bayesian modelling insight from the Romagnano Loc III rock shelter sequence (Trento, Italy)
Source: PLoS One. 2025 Sep 16;20(9):e0331392. doi: 10.1371/journal.pone.0331392 (PMC12440202; doi:10.1371/journal.pone.0331392)
Supplement: S9 Table — (PDF) [file pone.0331392.s009.pdf]

Supporting Information

The timing of the Castelnovisation of southwestern Europe: a Bayesian modelling insight from the Romagnano Loc III rock shelter sequence (Trento, Italy)

Salvador Pardo-Gordó, Alex Fontana, Vanessa Extrem-Membrado, Emilio Vacas-Fumero, Rossella Duches, Elisabetta Flor, Federica Fontana

Table: Radiometric dates related to sites used in the discussion section. The information about Romagnano is available in the main text.

Site	Region	Lab	BP	SD	Level	Type	Size	Material	13C	CN	Rank	Strata	Ref	Cal BCE (95%)
Shan Koba	Crimea	GrA-50242	7075	45	3 / 3	AMS	Sing.	Bone	-20.19	n.d	2	3	[1]	6058-5842
Shan Koba	Crimea	KIA-9572	7760	52	3 / 2	AMS	Sing.	Boar tooth	n.d	n.d	2	1	[1]	6686-6470
Shan Koba	Crimea	GrA-50241	7775	45	3 / 3-2	AMS	Sing.	Bone	-20.07	n.d	2	1	[1]	6686-6479
Shan Koba	Crimea	KIA-9573	7915	45	3 / 1	AMS	Sing.	Boar tooth	n.d	n.d	2	2	[1]	7034-6649
Shan Koba	Crimea	KIA-9571	8357	52	3 / 3	AMS	Sing.	*C. elaphus* tooth	n.d	n.d	2	3	[1]	7573-7194
Lapsi 7	Crimea	Ki-683	7620	230	D1-2	C	n.d	Charcoal	n.d	-	7	3	[2]	7061-6028
Lapsi 7	Crimea	Ki-704	8030	190	D	C	n.d	Charcoal	n.d	-	7	3	[2]	7475-6509
Lapsi 7	Crimea	Ki-957	8340	250	D	C	n.d	n.d	n.d	-	7	3	[2]	8165-6650
Lapsi 7	Crimea	Bln-1795.1	8570	75	D	C	n.d	Charcoal	n.d	-	7	3	[2]	7791-7479
Lapsi 7	Crimea	Ki-876	8680	250	D1	C	Bulk	Shells	n.d	-	6	3	[2]	8005-6603
Lapsi 7	Crimea	Bln-1795.2	8760	70	D1	C	n.d	Charcoal	n.d	-	7	3	[2]	8174-7597
Lapsi 7	Crimea	Ki-952	8870	120	D1	C	n.d	Charcoal	n.d	-	7	3	[2]	8279-7610
Lapsi 7	Crimea	Bln-1921	9085	100	D	C	n.d	Charcoal	n.d	-	7	3	[2]	8602-7960
Lapsi 7	Crimea	GrA-35703	8620	40	D	AMS	Sing.	Pomoideae	-26.52	-	4	2	[2]	7735-7582
Lapsi 7	Crimea	GrA-35704	8625	40	D	AMS	Sing.	*Ulmus* sp.	-24.51	-	4	2	[2]	7734-7584

Myrne	Crimea	GrA-37336	8240	45	PI Г24	AMS	Sing.	B. primigenius	-19.30	-	2	2	[2]	7459-7078
Myrne	Crimea	GrA-37335	8350	45	PIII B1	AMS	Sing.	Ungulate	-19.93	-	2	2	[2]	7535-7196
Myrne	Crimea	GrA-37337	8350	45	PII Б5	AMS	Sing.	*Equus* sp.	-21.13	-	2	2	[2]	7535-7196
Myrne	Crimea	GrA-37312	8475	45	PI Д22	AMS	Sing.	*Equus* sp.	-21.36	-	2	2	[2]	7590-7480
Konispol	Albania	Beta-67804	7630	140	6b (L.39)	C	n.d	Charcoal	n.d	-	7	2	[3]	7023-6095
Konispol	Albania	Beta-80000	7550	80	6d (L.41)	C	n.d	Charcoal	n.d	-	7	2	[3]	6570-6234
Konispol	Albania	Beta-67803	7510	90	6b (L.39)	C	n.d	Charcoal	n.d	-	7	2	[3]	6570-6092
Konispol	Albania	Beta-79999	7410	80	6c (L.42)	C	n.d	Charcoal	n.d	-	7	2	[3]	6420-6086
Konispol	Albania	Beta-80001	8900	180	6d (L.49)	C	n.d	Charcoal	n.d	-	7	3	[3]	8529-7591
Cverna Stijena	Montenegro	Beta-211504	7650	40	IV a	n.d	Sing.	Charcoal	n.d	-	7	2	[4]	6589-6432
Cverna Stijena	Montenegro	Beta-211503	7630	40	IV a	n.d	Sing.	Charcoal	n.d	-	7	2	[4]	6571-6420
Cverna Stijena	Montenegro	Beta-211505	8830	40	IV b	n.d	Sing.	Charcoal	n.d	-	7	2	[4]	8200-7750
Cverna Stijena	Montenegro	OxA-23344	7595	34	II	AMS	Sing.	Sus sp.	n.d	n.d	2	3	[4]	6498-6397
Cverna Stijena	Montenegro	OxA-23345	8870	37	IV a	AMS	Sing.	*Cervus elaphus*	n.d	n.d	2	3	[4]	8227-7829
Vruća Pećina	Montenegro	OxA-31133	8200	45	4/4	AMS	Sing.	Bone	-22.04	3.2	1	1	[5]	7344-7066
Odmut	Montenegro	Z-457*	7030	160	Ib	C	n.d	Charcoal	n.d	n.d	7	2	[6]	6222-5638
Odmut	Montenegro	SI-2227	7080	85	Ib	C	n.d	Charcoal	n.d	n.d	7	2	[6]	6080-5746
Odmut	Montenegro	SI-2220	7150	100	Ib	C	n.d	Charcoal	n.d	n.d	7	2	[6]	6231-5803
Odmut	Montenegro	Z-413*	7350	160	Ia	C	n.d	Charcoal	n.d	n.d	7	2	[6]	6502-5896
Odmut	Montenegro	Z-411*	7440	150	Ib	C	n.d	Charcoal	n.d	n.d	7	2	[6]	6588-6022
Odmut	Montenegro	OxA-32283	7757	38	XA	AMS	Sing.	Bone tool	-20.23	3.4	1	2	[5]	6648-6481
Odmut	Montenegro	SI-2221	7720	85	Ib	C	n.d	Charcoal	n.d	n.d	7	2	[6]	6773-6413
Odmut	Montenegro	OxA-35003	7770	40	Ia	AMS	Sing.	Bone tool	-20.5	3.2	1	2	[5]	6681-6481

Odmut	Montenegro	SI-2226	7790	70	Ib	C	n.d	Charcoal	n.d	n.d	7	2	[6]	6980-6460
Odmut	Montenegro	OxA-34966	7980	50	Ib	AMS	Sing.	Bone tool	-21.3	3.2	1	2	[5]	7050-6696
Odmut	Montenegro	OxA-35002	8207	39	?	AMS	Sing.	Bone tool	-20.3	3.2	1	3	[5]	7342-7072
Odmut	Montenegro	SI-2224	8590	100	Ia / Ib	C	n.d	Charcoal	n.d	n.d	7	2	[6]	7954-7378
Odmut	Montenegro	SI-2228	9135	80	Ib	C	n.d	Charcoal	n.d	n.d	7	2	[6]	8602-8231
Grotta dell'Uzzo	Italy	MAMS-16238	7957	25	F-12	AMS	Sing.	*Mysticeti* sp.	-12.9	3.2	6	1	[7]	6444-6011
Grotta dell'Uzzo	Italy	P-2734	7910	70	F-13/14	C	n.d	Charcoal	n.d	n.d	7	1	[8]	7044-6641
Grotta dell'Uzzo	Italy	MAMS-40719	7809	26	U	AMS	Sing.	Human Bone	n.d	n.d	6	3	[9]	6694-6572
Grotta dell'Uzzo	Italy	OxA-V-2364-43	7753	36	F-12	AMS	Sing.	Human Bone	-16.2	3.1	6	2	[7]	6503-6222
Grotta dell'Uzzo	Italy	OxA-13662	7744	33	F-12	AMS	Sing.	*Phorcus turbinatus*	n.d	n.d	6	1	[10]	6224-5932
Grotta dell'Uzzo	Italy	KIA-36034	7730	80	F-14	AMS	Sing.	*Epinephelus* sp.	-12.4	3.2	6	1	[7]	6281-5841
Grotta dell'Uzzo	Italy	MAMS-40709	7713	26	F-15	AMS	Sing.	Human Bone	n.d	n.d	6	2	[11]	6633-6469
Grotta dell'Uzzo	Italy	MAMS-40726	7471	26	F-14	AMS	Sing.	Human Bone	n.d	n.d	6	2	[11]	6416-6245
Grotta dell'Uzzo	Italy	KIA-36032	7175	45	F-11	AMS	Sing.	*Epinephelus* sp.	-10.2	3.3	6	1	[7]	5661-5370
Latronico 3	Italy	Rome-453	8024	100	A 63-64	C	n.d	Charcoal	n.d	-	7	1	[12]	7310-6647
Latronico 3	Italy	Rome-449	7800	90	A 55	C	n.d	Charcoal	n.d	-	7	1	[12]	7031-6458
Latronico 3	Italy	Rome-446	7620	90	D 43	C	n.d	Charcoal	n.d	-	7	1	[20]	6644-6257
Latronico 3	Italy	Rome-448	7570	90	B 53-54	C	n.d	Charcoal	n.d	-	7	1	[20]	6594-6237
Latronico 3	Italy	Rome-445	7420	90	D 41-42	C	n.d	Charcoal	n.d	-	7	1	[20]	6431-6083

Latronico 3	Italy	Rome-447	7400	90	B 52	C	n.d	Charcoal	n.d	-	7	1	[20]	6424-6077
Latronico 3	Italy	Rome-451	7160	80	A 58	C	n.d	Charcoal	n.d	-	7	1	[20]	6224-5851
Latronico 3	Italy	Rome-450	7045	90	A 57	C	n.d	Charcoal	n.d	-	7	1	[20]	6065-5740
Latronico 3	Italy	Rome-452	6979	90	A 59-60	C	n.d	Charcoal	n.d	-	7	1	[20]	6024-5674
Terragne di Manduria	Italy	Beta-67093	7260	70	US 4-5	AMS	Sing.	*Bos* sp.	n.d	n.d	2	1	[21]	6330-5988
Lama Lite	Italy	R-1394	6620	80	T4	C.	n.d	Charcoal	n.d	-	7	1	[22]	5711-5387
Lama Lite	Italy	R-394	6620	80	6	C	n.d	Charcoal	n.d	-	7	1	[23]	5711-5387
Piazzana	Italy	Rome-400	7330	85	3A	C	n.d	Charcoal	n.d	-	7	1	[23]	6388-6030
Riparo Gaban	Italy	KIA-10365	8323	63	FA	AMS	n.d	Charcoal	n.d	-	7	1	[24]	7531-7178
Riparo Gaban	Italy	KIA-10364	7971	42	FA	AMS	n.d	Charcoal	n.d	-	7	1	[24]	7045-6697
Riparo Gaban	Italy	KIA-10367	7902	55	FA	AMS	n.d	Charcoal	n.d	-	7	1	[24]	7036-6644
Riparo Gaban	Italy	KIA-10366	7725	49	FA	AMS	n.d	Charcoal	n.d	-	7	1	[24]	6643-6467
Riparo Gaban	Italy	KIA-10363	6968	41	E	AMS	n.d	Charcoal	n.d	-	7	1	[24]	5977-5742
Mondeval de Sora	Italy	OxA-7488	7425	55	Burial	AMS	Sing.	Human Bone	-19.5	n.d	2	1	[25]	6425-6091
Mezzocorona-Borgonuovo	Italy	ETH-15980	6005	75	145	AMS	Sing.	Human Bone	-21	n.d	2	2	[26]	5206-4713
Mezzocorona-Borgonuovo	Italy	ETH-15981	6170	70	145	AMS	Sing.	Human Bone	-22	n.d	2	2	[26]	5306-4938
Mezzocorona-Borgonuovo	Italy	ETH-15984	6410	75	151	AMS	Sing.	*Cervus elaphus*	-24.1	n.d	2	2	[26]	5515-5216
Mezzocorona-Borgonuovo	Italy	UtC-7202	6210	60	145	AMS	Sing.	Human Bone	-23.8	n.d	2	2	[26]	5307-5008
Mezzocorona-Borgonuovo	Italy	UtC-7201	6380	50	145	AMS	Sing.	Human Bone	-23.8	n.d	2	2	[26]	5474-5222
Mezzocorona-Borgonuovo	Italy	MAMS-56784	6538	29	145	AMS	Sing.	Human Bone	n.d	3.24	1	2	[27]	5612-5409

Mezzocorona-Borgonuovo	Italy	KIA-12446	7797	43	148	AMS	Sing.	*Cervus elaphus*	-21.1	n.d	2	2	[27]	6742-6481
Baume Montclus	France	Beta-255115	7770	50	L. 15	AMS	Sing.	*Cervus* sp.	n.d	n.d	2	3	[28]	6687-6475
Baume Montclus	France	Beta-432166	7760	30	L. 16	AMS	Sing.	*Vitis* sp.	n.d	-	1	3	[29]	6648-6498
Baume Montclus	France	Beta-253166	7670	50	L. 16	AMS	Sing.	Bone	n.d	n.d	2	3	[28]	6636-6431
Baume Montclus	France	Ly-542	7540	160	L. 16	AMS	Sing.	Bone	n.d	n.d	2	3	[21]	6748-6031
Baume Montclus	France	Beta-253164	7320	50	L. 14B	AMS	Sing.	Bone	n.d	n.d	2	1	[20]	6351-6064
Baume Montclus	France	Beta-253163	7190	50	L. 13B	AMS	Sing.	*Capreolus capreolus*	n.d	n.d	2	1	[20]	6219-5926
Baume Montclus	France	Beta-253162	7170	50	L. 12A	AMS	Sing.	Bone	n.d	n.d	2	1	[20]	6216-5917
Baume Montclus	France	Ly-496	7020	140	L. 14	C	Bulk	Sediment	n.d	-	7	1	[21]	6216-5639
Baume Montclus	France	Beta-253161	6990	40	L. 10	AMS	Sing.	*Sus scrofa*	n.d	n.d	2	1	[20]	5982-5755
Baume Montclus	France	MC-728	6880	100	L. 8	C	Bulk	Charcoal	n.d	-	7	1	[21]	5984-5622
Baume Montclus	France	Beta-253160	6660	40	L. 7	AMS	Sing.	*Capreolus capreolus*	n.d	n.d	2	1	[20]	5653-5483
Baume Montclus	France	Ly-495	6440	230	L. 14	C	Bulk	Sediment	n.d	-	7	1	[21]	5794-4845
Baume Montclus	France	MC-729	6440	230	L. 13	C	Bulk	Charcoal	n.d	-	7	1	[21]	5794-4845

Baume Montclus	France	Ly-494	6230	150	L. 8	C	Bulk	Charcoal	n.d	-	7	1	[21]	5478-4803
Font-des-Pigeons	France	LTL1-5949A	7573	60	L. 19	AMS	Sing.	*Phyllyrea* sp. / *Rhamnus alaternus*	n.d	-	4	1	[20]	6570-6251
Font-des-Pigeons	France	LTL-15419A	7382	50	L.18	AMS	Sing.	*Pistacea* sp. (seed)	n.d	-	1	1	[20]	6382-6086
Font-des-Pigeons	France	LTL-15416A	7369	55	L. 18	AMS	Sing.	*Pistacea* sp. (seed)	n.d	-	1	1	[20]	6377-6082
Font-des-Pigeons	France	LTL-15786A	7357	50	L. 18	AMS	Sing.	*Phyllyrea* sp. / *Rhamnus alaternus*	n.d	-	4	1	[20]	6373-6077
Font-des-Pigeons	France	Ly-2832	7290	130	L. 19	C	n.d	Charcoal	n.d	-	7	1	[22]	6426-5916
Font-des-Pigeons	France	Ly-2830	7260	120	L. 18	C	n.d	Charcoal	n.d	-	7	1	[22]	6397-5901
Font-des-Pigeons	France	LTL-15785A	6633	50	L. 18	AMS	Sing.	*Arbutus unedo*	n.d	-	4	1	[20]	5629-5480
Font-des-Pigeons	France	LTL-15782A	6539	45	L. 18	AMS	Sing.	*Phyllyrea* sp. / *Rhamnus alaternus*	n.d	-	4	1	[20]	5616-5381
Font-des-Pigeons	France	LTL-15950A	6400	45	L. 19	AMS	Sing.	*Pinus* sp.	n.d	-	4	1	[20]	5475-5230
Font-des-Pigeons	France	Beta-267434	6250	40	L. 18	AMS	Sing.	*Cerealia* sp. (seed)	n.d	-	1	1	[20]	5312-5066
Grande-Rivoire	France	Beta-282248	7790	45	US 30/32	AMS	Sing.	Bone	n.d	n.d	2	3	[23]	6696-6477

Grande-Rivoire	France	GrA-25066	7435	45	6.66 B3	AMS	Sing.	Bone	n.d	n.d	2	1	[22]	6417-6226
Grande-Rivoire	France	Beta-255119	7310	40	US 25/27	AMS	Sing.	Bone	n.d	n.d	2	1	[22]	6235-6073
Mourre du Sève	France	Lyon-148	7730	60	E2	AMS	Sing.	*Quercus* sp.	n.d	-	4	1	[24]	6681-6453
Mourre du Sève	France	Lyon-149	7640	65	E2	AMS	Sing.	*Quercus* sp.	n.d	-	4	1	[24]	6638-6396
Mourre du Sève	France	Lyon-150	7414	60	E2	AMS	Sing.	*Quercus* sp.	n.d	-	4	1	[24]	6420-6089
Roquemissou	France	Lyon-12839 / SacA-45094	7545	40	E	AMS	Sing.	Bone	n.d	n.d	2	1	[25]	6469-6260
Roquemissou	France	Beta-363427	7500	40	E	AMS	Sing.	*Bos* sp.	-21.3	n.d	2	1	[25]	6436-6246
Roquemissou	France	Lyon-11260 / SacA-3726	7150	35	E	AMS	Sing.	*Corylus avellana*	n.d	-	1	1	[25]	6071-5926
Roquemissou	France	Beta-398965	7140	30	E	AMS	Sing.	*Capreolus capreolus*	-22.3	n.d	2	1	[25]	6067-5926
Roquemissou	France	Lyon-12841 / SacA-45096	7000	45	E	AMS	Sing.	Bone	n.d	n.d	2	1	[25]	5986-5760
Roquemissou	France	Beta-363428	6630	40	E	AMS	Sing.	*Sus* sp.	-19.3	n.d	2	1	[25]	5625-5482
Abric de la Falguera	Spain (East)	AA-2295	7410	70	VII	AMS	Sing.	*Olea* sp. (seed)	n.d	-	1	1	[26]	6419-6087
Abric de la Falguera	Spain (East)	Beta-171909	7280	40	VII	AMS	Sing.	*Pinus halepensis*	n.d	-	4	1	[26]	6229-6065
Abric de la Falguera	Spain (East)	AA-59519	7526	44	VIII	AMS	Sing.	*Pinus halepensis* (bract)	n.d	-	1	1	[26]	6460-6251

Barranc de la Fontanella	Spain (East)	Beta-573649	7830	30	IIa	AMS	Sing.	*Bos* sp.	n.d	n.d	2	1	[27]	6769-6591
Benàmer	Spain (East)	CNA-680	7490	50	I	AMS	Bulk	Pollen	n.d	-	3	1	[28]	6434-6241
Benàmer	Spain (East)	Beta-287331	7480	40	I	AMS	Bulk	Pollen	n.d	-	3	1	[28]	6425-6243
Casa Corona	Spain (East)	Beta-272856	7070	40	Burial 1	AMS	Sing.	Human	n.d	n.d	2	1	[29]	6024-5844
Casa Corona	Spain (East)	OxA-V-2392-92	7116	32	Burial 2	AMS	Sing.	Human	n.d	n.d	2	1	[29]	6064-5915
Cueva Blanca	Spain (East)	Beta-288287	7610	40	1b/2sup	AMS	Sing.	*Pinus halepensis*	n.d	-	4	1	[30]	6570-6396
Cueva de la Cocina	Spain (East)	PSU-5320	7040	20	Pericot 1943-4	AMS	Sing.	*Cervus elaphus*	-20.1	2.9	1	1	[31]	5987-5847
Cueva de la Cocina	Spain (East)	PSU-5321	7160	25	Pericot 1943-9	AMS	Sing.	*Capra pyrenaica*	-20.2	2.9	1	1	[31]	6070-5988
Cueva de la Cocina	Spain (East)	PSU-5608	7285	25	Pericot 1942-4	AMS	Sing.	*Cervus elaphus*	-20.2	2.8	8	1	[31]	6222-6075
Cueva de la Cocina	Spain (East)	PSU-5322	7310	25	Pericot 1942-8	AMS	Sing.	*Capra pyrenaica*	-20.7	3	1	1	[31]	6227-6083
Cueva de la Cocina	Spain (East)	PSU-5323	6590	25	Pericot 1941-1	AMS	Sing.	*Capra pyrenaica*	-19.8	2.9	1	1	[31]	5613-5479
Cueva de la Cocina	Spain (East)	Beta-512548	6940	30	García 2018-1424	AMS	Sing.	*Capra pyrenaica*	-19	3.3	1	1	[31]	5894-5732
Cueva de la Cocina	Spain (East)	Beta-512549	6760	30	García 2015-1424	AMS	Sing.	*Capra pyrenaica*	-20.2	3.3	1	1	[31]	5720-5625
Cueva de la Cocina	Spain (East)	Beta-512550	6910	30	García 2015-1147	AMS	Sing.	*Capra pyrenaica*	-19.6	3.3	1	1	[31]	5881-5724
Cueva de la Cocina	Spain (East)	Beta-426850	7380	30	García 2015-1154	AMS	Sing.	*Quercus ilex*	-36.4	-	4	1	[31]	6376-6088
Cueva de la Cocina	Spain (East)	Beta-453590	6930	30	García 2015-1021	AMS	Sing.	Acorn	-23.3	-	1	1	[31]	5887-5731

Cueva de la Cocina	Spain (East)	UCIAMS-174945	6705	35	García 2015-1078	AMS	Sing.	*Capra pyrenaica*	-20.5	3.23	1	1	[31]	5714-5556
Cueva de la Cocina	Spain (East)	Beta-599654	6760	30	García 2016-1223	AMS	Sing.	Pine cone	-27.9	-	1	1	[31]	5720-5625
Cueva de la Cocina	Spain (East)	Beta-599655	6880	30	García 2016-1230	AMS	Sing.	Pine cone	-24.3	-	1	1	[31]	5841-5674
Cueva de la Cocina	Spain (East)	Beta-599656	6980	30	García 2018-1404	AMS	Sing.	Pine cone	-20.7	-	1	1	[31]	5978-5757
Cueva de la Cocina	Spain (East)	Beta-599657	6970	30	García 2018-1406	AMS	Sing.	Pine cone	-27.8	-	1	1	[31]	5973-5751
Cueva de la Cocina	Spain (East)	Beta-599658	6770	30	García 2015-1020	AMS	Sing.	Pine cone	-21.9	-	1	1	[31]	5722-5627
Cueva de la Cocina	Spain (East)	UCIAMS-147347	7415	35	Pericot 1941-11	AMS	Sing.	*Capra pyrenaica*	n.d	n.d	2	1	[32]	6396-6112
Cueva de la Cocina	Spain (East)	UCIAMS-147348	7905	40	Pericot 1941-13	AMS	Sing.	*Capra pyrenaica*	n.d	n.d	2	3	[32]	7032-6647
Cueva de la Cocina	Spain (East)	UCIAMS-147346	6970	35	Pericot 1941-3	AMS	Sing.	*Cervus elaphus*	n.d	n.d	2	1	[32]	5975-5747
Cueva de la Cocina	Spain (East)	UCIAMS-145194	7300	30	Pericot 1941-6	AMS	Sing.	*Cervus elaphus*	n.d	n.d	2	1	[32]	6226-6078
Cueva de la Cocina	Spain (East)	UCIAMS-145195	7475	25	Pericot 1941-8	AMS	Sing.	*Capra pyrenaica*	n.d	n.d	2	1	[32]	6419-6246
Cueva de la Cocina	Spain (East)	Beta-267438	7350	40	Pericot 1945-12	AMS	Sing.	*Capra pyrenaica*	n.d	n.d	2	1	[33]	6364-6077
Cueva de la Cocina	Spain (East)	Beta-267439	6760	40	Pericot 1945-13	AMS	Sing.	*Capra pyrenaica*	n.d	n.d	2	1	[33]	5727-5573
Cueva de la Cocina	Spain (East)	Beta-267440	7610	40	Pericot 1945-17	AMS	Sing.	*Capra pyrenaica*	n.d	n.d	2	1	[33]	6570-6396

Cueva de la Cocina	Spain (East)	Beta-267435	6840	50	Pericot 1945-6	AMS	Sing.	*Capra pyrenaica*	n.d	n.d	2	1	[33]	5831-5634
Cueva de la Cocina	Spain (East)	Beta-267436	7080	50	Pericot 1945-8	AMS	Sing.	*Capra pyrenaica*	n.d	n.d	2	1	[33]	6061-5843
Cueva de la Cocina	Spain (East)	Beta-267437	7050	50	Pericot 1945-10	AMS	Sing.	*Capra pyrenaica*	n.d	n.d	2	1	[33]	6023-5803
Cueva de la Cocina	Spain (East)	PSU-4429	7135	25	Pericot 1941-2	AMS	Sing.	Human	-18.8	3.27	1	1	[34]	6064-5926
Cueva de la Cocina	Spain (East)	UCIAMS-145198	6985	25	Fortea H B5-2	AMS	Sing.	*Capra pyrenaica*	n.d	n.d	2	1	[35]	5980-5782
Cueva de la Cocina	Spain (East)	UCIAMS-145196	7455	25	Fortea H H3-5	AMS	Sing.	*Cervus elaphus*	n.d	n.d	2	1	[35]	6396-6241
Cueva de la Cocina	Spain (East)	UCIAMS-145197	7710	30	Fortea H H4-6	AMS	Sing.	*Capra pyrenaica*	n.d	n.d	2	1	[35]	6636-6467
Cueva de la Cocina	Spain (East)	UCIAMS-174943	7400	30	Pericot 1943-8/9	AMS	Sing.	Human	-18.1	3.3	1	1	[36]	6385-6102
Cueva de la Cocina	Spain (East)	UCIAMS-174147	7375	25	Pericot 1942-1	AMS	Sing.	Human	-19.3	3.26	1	1	[36]	6371-6087
Cueva de la Cocina	Spain (East)	Beta-618258	7120	30	Sarrión 1974	AMS	Sing.	Human	-18.8	3.3	1	3	[36]	6063-5919
Cueva de la Cocina	Spain (East)	Beta-618257	7090	30	Sarrión 1974	AMS	Sing.	Human	-18.2	3.2	1	3	[36]	6026-5893
El Collao	Spain (East)	Beta-337186	7820	30	I	AMS	Sing.	*Cervus Elaphus*	n.d	n.d	2	1	[37]	6749-6571
El Collao	Spain (East)	UBA-27478	7660	44	I	C	Sing.	*Cervus Elaphus*	n.d	n.d	2	1	[37]	6593-6434
El Collao	Spain (East)	Ua-72903	7484	38	Surface	AMS	Sing.	*Cervus Elaphus*	n.d	n.d	2	3	[38]	6426-6245
Tossal de la Roca	Spain (East)	Gif-6897	7560	80	I / Ext.	C	Bulk	Bone	n.d	n.d	3	1	[39]	6571-6238

Tossal de la Roca	Spain (East)	Gif-6898	7660	80	I / Ext.	C	Bulk	Bone	n.d	n.d	3	1	[39]	6652-6381
Costa do Pereiro	Portugal	Wk-17026	7327	42	C1	AMS	Sing.	*Cervus* sp.	n.d	n.d	2	1	[40]	6334-6071
Cova da Beleia	Portugal	Beta-464381	7330	30	Phase II	AMS	Sing.	Charcoal	n.d	n.d	7	1	[41]	6236-6081
Prazo	Portugal	GrA-18787	6950	50	SU 4a	AMS	Sing.	Seed	n.d	n.d	1	2	[42]	5975-5730
Prazo	Portugal	OxA-24779	7792	34	SU 4a	AMS	Sing.	Charcoal	-24.9	-	7	2	[42]	6689-6507
Prazo	Portugal	GrN-26400	6710	50	SU 4a	C	n.d	Charcoal	n.d	-	7	2	[42]	5720-5535
Samouqueira	Portugal	Beta-452075	7120	30	Lev. 3	AMS	Sing.	Bone	-20.6	n.d	2	1	[43]	6063-5919
Samouqueira	Portugal	ICEN-1233	7590	60	Lev. 3	C	Sing.	*Thais haemostoma*	0.39	n.d	6	1	[43]	5993-5650
Samouqueira	Portugal	ICEN-1232	7550	60	Lev. 3	C	Sing.	*Patella* sp.	0	-	6	1	[43]	5966-5622
Samouqueira	Portugal	ICEN-729	7520	60	Lev. 3	C	Bulk	*Patella* sp.	-0.06	-	6	1	[40]	5953-5595
Samouqueira	Portugal	OxA-36994	6995	36	Lev. 2	AMS	Sing.	*Patella* sp.	n.d	-	6	3	[44]	5435-5098
Samouqueira	Portugal	OxA-36936	7015	38	Lev. 2	AMS	Sing.	Human	-15.2	3.3	6	3	[43]	5456-5127
Samouqueira	Portugal	TO-130	6370	70	Lev. 2	C	Sing.	Human	-15.3	n.d	6	3	[45]	5475-5216
Vale de Romeiras	Portugal	ICEN-150	7390	80	Middle 2	C	Bulk	Shell	n.d	-	6	1	[45]	6193-5475
Vale de Romeiras	Portugal	ICEN-144	7130	110	Middle 2	C	Bulk	Bone	n.d	n.d	3	1	[45]	6228-5774
Vale de Romeiras	Portugal	Ua-46972	7640	55	Burial 19	AMS	Sing.	Human	-20.2	3.2	1	1	[46]	6598-6408
Vale Marim	Portugal	Beta-417015	7020	30	2A	AMS	Sing.	*Pinus* sp.	n.d	-	4	1	[47]	5987-5833
Vale Marim	Portugal	Beta-417016	7180	30	2A	AMS	Bulk	Charcoal	n.d	-	7	1	[47]	6076-5990
Vale Marim	Portugal	Beta-373853	7170	40	2A	AMS	Sing.	*Arbutus unedo*	n.d	-	4	1	[47]	6086-5923

References

1. Biagi P, Khlopachev GA, Kiosak D. The radiocarbon chronology of Shan-Koba Rock-Shelter, a late palaeolithic and mesolithic sequence in the crimean mountains (Ukraine). Diadora. 2014;28: 7–20.

2. Biagi P, Kiosak. The Mesolithic of the northwestern Pontic region New AMS dates for the origin and spread of the blade and trapeze industries in southeast Europe. Eurasia Antiqua. 2010;16: 21–41.

3. Harrold FB, Russell N, Wickens J. The Mesolithic of Konispol cave, Albania. Iliria. 2016;49: 7–33.

4. Mercier N, Rink WJ, Rodriguez K, Morley MW, Whallow R. Radiometric Dating of the Crvena Stijena Sequence. In: Whallow R, editor. Crvena Stijena in Cultural and Ecological Context: Multidisciplinary Archaeological Research in Montenegro. National Museum of Montenegro Montenegrin Academy of Sciences and Arts; 2017. pp. 140–149.

5. Borić D, Borovinić N, Duričič L, Bulatović J, Gerometta K, Filipovič D, et al. Spearheading into the Neolithic: Last Foragers and First Farmers in the Dinaric Alps of Montenegro. European Journal of Archaeology. 2019;22 (4): 470–498.

6. Cristiani E, Borić D. Mesolithic harpoons from Odmut, Montenegro: Chronological, contextual, and techno-functional analyses. Quaternary International. 2016;423: 166–192.

7. Mannino MA, Talamo S, Tagliacozzo A, Fiore I, Nehlich O, Piperno M, et al. Climate-driven environmental changes around 8,200 years ago favoured increases in cetacean strandings and Mediterranean hunter-gatherers exploited them. Scientific Reports. 2015;5: 16288.

8. Meulengracht A, McGovern P, Lawn B. University od pennsylvania radiocarbon dates XXI. Radiocarbon. 1981;23: 227–240.

9. Yu H, Loosdrecht MS van de, Mannino MA, Talamo S, Rohrlach AB, Childebayeva A, et al. Genomic and dietary discontinuities during the Mesolithic and Neolithic in Sicily. iScience. 2022;25: 104244. doi:https://doi.org/10.1016/j.isci.2022.104244

10. Mannino MA, Thomas KD, Leng MJ, Piperno M, Tusa S, Tagliacozzo A. Marine resources in the Mesolithic and Neolithic at teh Grotta dell'Uzzo (Sicily): Evidence from isotope analyses of marine shells. Archaeometry. 2007;49: 117–133. doi:10.1111/j.1475-4754.2007.00291.x

11. Loosdrecht MS van de. Archaeogenetic perspectives on the hunter-gatherers and prehistoric farmers of the Mediterranean. PhD of Philosophy, Friedrich Schiller University Jena. 2021.

12. Skeates R, Whitehouse R. New radiocarbon Dates for prehistoric italy, 2. The Accordia Research Papers. 1997;6: 179–191.

13. Natali E, Forgia V. The beginning of the Neolithic in Southern Italy and Sicily. Quaternary International. 2018;470: 253–269.

14. Cremonesi RG. Le Néolithique ancien de Toscane et de l'Archipel toscan. Bulletin de la Société Préhistorique Française. 2001;98–3: 423–429.

15. Boschian G, Mallegni F, Tozzi C. The epigravetian and mesolithic site of Fredian shelter (in Tuscany). Quaternary Nova. 1995;1995: 45–80.

16. Kozlowski SK, Dalmeri G. Riparo Gaban: the Mesolithic layers. Preistoria Alpina. 2000;36: 3–42.

17. Bronk Ramsey C, Higham T, Owen D, Pike AW, Hedges REM. Radiocarbon dates from the Oxford AMS Sustem: Archaeometry Datelist 31. Archaeometry. 2002;44 (3): 1–149.

18. Dalmeri G, Mottes E, Nicolis F. The Mesolithic burial of Mezzocorona-Burgonuovo (Trento): some preliminary comments. Preistoria Alpina. 2001;34: 129–138.

19. Sparacello VS, Mottes E, Dori I, Posth C, Nicolis F. A history of violence in the Mesolithic female skeleton from Mezzocorona-Borgonuovo (Trento, northeastern Italy). Quaternary Science Reviews. 2023;311: 108149. doi:doi.org/10.1016/j.quascirev.2023.108149

20. Binder D, Battentier J, Delhon C, Sénépart I. In pursuit of a missing transition: the Mesolithic and Neolithic radiocarbon chronology at La Font-aux-Pigeons rockshelter. Antiquity. 2017;91: 605–620. doi:10.15184/aqy.2017.65

21. Defranould E, Perrin T. Evolutionary Dynamics of Armatures in Southern France in the Late Mesolithic and Early Neolithic. Open Archaeology. 2022;8(1): 905–924. doi:10.1515/opar-2022-0261

22. Marchand G, Perrin T. Why this revolution? Explaining the major technical shift in Southwestern Europe during the 7th millennium cal. BC. Quaternary International. 2017;428: 73–85. doi:10.1016/j.quaint.2015.07.059

23. Nicod P-Y, Perrin T, Brochier J-L, Chaix L, Marquebielle B, Picavet R, et al. Continuités et ruptures culturelles entre chasseurs mésolithiques et chasseurs néolithiques en Vercors: analyse préliminaire des niveaux du Mésolithique récent et du Néolithique ancien… In: Perrin T, Sénépart I, Cauliez J, Thirault É, Bonnardin S, editors. 9e RMPR (Saint-Georges-de-Didonne 2010) — Dynamismes et rythmes évolutifs des sociétés de la Préhistoire Récente. Tolouse: Archives d'Écologie Préhistorique; 2012. pp. 13–32.

24. Binder D, Sénépart I. Derniers chasseurs et premiers paysans de Vaucluse. Mésolithique et Néolithique ancien: 7000-4700 av. J.-C. In: Buisson-Catil J, Guilcher A, Hussy C, Pagni M, Olive M, editors. Vaucluse Préhistorique Le territoire, les hommes, les cultures et les site. Avignon: Editions A. Barthélemy; 2004. pp. 131–162.

25. Perrin T, Manen C, Valdeyron N, Guilaine J. Beyond the sea… The Neolithic transition in the southwest of France. Quaternary International. 2018;470: 318–332.

26. García-Puchol O, Aura JE. El abric de la Falguera (Alcoi, Alacant): 8.000 años de ocupación humana en la cabecera del río de Alcoi. Alcoy: Ayuntamiento de Alcoy; 2006.

27. Roman Monroig D, Domingo Sanz I, Bergadà Zapata MM, Lloveras L, Nadal J. La Balma del Barranc de La Fontanella (Vilafranca, castelló) y sus implicaciones en el conocimiento del Mesolítico Geométrico del Mediterráneo ibérico y el Valle del Ebro. Complutum. 2023;34(1): 9–30. doi:10.5209/cmpl.88937

28. Torregrosa Giménez P, Jover Maestre FJ, López Seguí E. Benàmer (Muro de Alcoi). Mesolíticos y neolítico en tierras meridionales valencianas. Valencia: Museu de Prehistòria; 2011.

29. Fernández López JF-L, Salazar-García DC, Subirà-Galdacano ME, Roca de Togores Muñoz C, Gómez-Puche M, Richards MP, et al. Late Mesolithic burials at Casa Corona (Villena, Spain): direct radiocarbon and palaeodietary evidence of the last forager populations in Eastern Iberia. Journal of Archaeological Science. 2013;40: 671–680.

30. Mingo A, Barba J, Uzquiano Ollero P, Casas M, Benito Calvo A, Yravedra Sainz de los Terreros J, et al. El yacimiento mesolítico de Cuva Blanca (Hellín, Albacete): 6 años de investigación multidisciplinar. In: Gamo B, Sanz R, editors. I reunión científica de Arqueología de Albacete. Albacete: Instituto de Estudios Albacetenses "Don Juan Manuel; 2016. pp. 51–66.

31. García-Puchol O, McClure SB, Juan-Cabanilles J, Cortell-Nicolau A, Diez-Castillo A, Benito JLP, et al. A multi-stage Bayesian modelling for building the chronocultural sequence of the Late Mesolithic at Cueva de la Cocina (Valencia, Eastern Iberia). Quaternary International. 2023;677–678: 18–35. doi:https://doi.org/10.1016/j.quaint.2023.05.015

32. García Puchol O, McClure SB, Juan-Cabanilles J, Diez-Castillo AA, Bernabeu-Aubán J, Martí-Oliver B, et al. Cocina Cave Revisited: Bayesian radiocarbon chronology for the last hunter-gatherers and first farmers in Eastern Iberia. Quaternary International. 2017.

33. Juan-Cabanilles J, García Puchol O. Rupture et continuité dans la néolithisation du versant méditerranéen de la péninsule Ibérique: mise à l'épreuve du modèle du dualité culturelle. Transition, ruptures et continuité durant la Préhistoire Actes du XXVIIe Congrès Préhistorique de France, Bordeaux-Les Eyzies. 2013;31: 405–417.

34. Olalde I, Mallick S, Patterson N, Rohland N, Villalba-Mouco V, Silva M, et al. The genomic history of the Iberian Peninsula over the past 8000 years. Science. 2019;363: 1230. doi:10.1126/science.aav4040

35. Pardo-Gordó S, García Puchol O, Diez Castillo A, McClure SB, Juan Cabanilles J, Pérez Ripoll M, et al. Taphonomic processes inconsistent with indigenous Mesolithic acculturation during the transition to the Neolithic in the Western Mediterranean. Quaternary International. 2018. doi:https://doi.org/10.1016/j.quaint.2018.05.008

36. McClure SB, Pérez Fernández Á, García Puchol O, Juan Cabanilles J. Mesolithic human remains at Cueva de la Cocina: Insights from bioarchaeology and geochemistry. Quaternary International. 2023;677–678: 36–50. doi:10.1016/j.quaint.2023.07.001

37. Fernández-López de Pablo J. The timing of postglacial coastal adaptations in Eastern Iberia: A Bayesian chronological model for the El Collado shell midden (Oliva, Valencia, Spain). Quaternary International. 2016;407: 94–105.

38. Gómez-Puche M, Fernández-López de Pablo J. Spatiotemporal patterns on the appearance of the first trapeze industries in the Late Mesolithic of the Iberian Peninsula. Radiocarbon. 2024/02/14 ed. 2024;66: 59–100. doi:10.1017/RDC.2024.6

39. Martí B, Aura Tortosa JE, García Puchol O, Fernández-López de Pablo J. El mesolítico geométrico de tipo “cocina” en el País Valenciano. In: Utrilla P, Montes L, editors. El mesolítico geométrico en la Península Ibérica. Zaragoza: Universidad de Zaragoza; 2009. pp. 205–258.

40. Carvalho AF. O Mesolítico final em Portugal. In: Utrilla P, Montes L, editors. El mesolítico geométrico en la Península Ibérica. Zaragoza: Universidad de Zaragoza; 2009. pp. 33–68.

41. Sousa AC, Gibaja Bao J, Mazzuco N, Miranda M, Tereso JPV, Oliveira C, et al. Clay combustion structures in early Mesolithic at Cova da Baleia (Mafra, Portugal): Approaches to their functionality. Journal of Archaeological Science: Reports. 2017. doi:10.1016/j.jasrep.2017.10.049

42. Monteiro-Rodrigues S. Novas datações pelo carbono-14 para as ocupações holocénicas do Prazo (Freixo de Numão, Vila Nova de Foz Côa, Norte de Portugal). Estudos do Quaternário/Quaternary Studies. 2012;1.

43. Soares J, Tavares da Silva C. Living in the southwest Portuguese coast during the Late Mesolithic: The case study of Vale Marim I. Journal of Archaeological Science: Reports. 2017. doi:10.1016/j.jasrep.2017.11.019

44. Jackes M, Lubell D, Meiklejohn C, Schulting R, Arias Cabal P. New analyses and dating of partial skeletons from Samouqueira I, Portugal. Mesolithic Miscellany. 2019;27: 3–23.

45. Zilhão J. From the mesolithic to the neolithic in the Iberian peninsular. In: Douglas PT, editor. Europe’s first farmers. Cambridge: The press syndicate of the University of Cambrigde; 2000. pp. 144–182.

46. Peyroteo-Stjerna R. On Death in the Mesolithic or the Mortuary Practices of the Last Hunter-Gatherers of the South-Western Iberian Peninsula, 7th–6th Millennium BCE. Uppsala University. 2016.

47. Soares J, Mazzucco N, Tavares da Silva C. Marine adaptations in the Late Mesolithic of the Portuguese southwest coast: use-wear analysis of the lithic industry of Vale Marim I. Revista Portuguesa de Arqueologia. 2017;20: 31–44.
